# Coral Reef Resilience, Tipping Points and the Strength of Herbivory

**DOI:** 10.1038/srep35817

**Published:** 2016-11-02

**Authors:** Sally J. Holbrook, Russell J. Schmitt, Thomas C. Adam, Andrew J. Brooks

**Affiliations:** 1 Department of Ecology, Evolution and Marine Biology, University of California Santa Barbara, Santa Barbara, CA, 93106, USA; 2 Coastal Research Center, Marine Science Institute, University of California Santa Barbara, Santa Barbara, CA, 93106, USA

## Abstract

Coral reefs increasingly are undergoing transitions from coral to macroalgal dominance. Although the functional roles of reef herbivores in controlling algae are becoming better understood, identifying possible tipping points in the herbivory-macroalgae relationships has remained a challenge. Assessment of where any coral reef ecosystem lies in relation to the coral-to-macroalgae tipping point is fundamental to understanding resilience properties, forecasting state shifts, and developing effective management practices. We conducted a multi-year field experiment in Moorea, French Polynesia to estimate these properties. While we found a sharp herbivory threshold where macroalgae escape control, ambient levels of herbivory by reef fishes were well above that needed to prevent proliferation of macroalgae. These findings are consistent with previously observed high resilience of the fore reef in Moorea. Our approach can identify vulnerable coral reef systems in urgent need of management action to both forestall shifts to macroalgae and preserve properties essential for resilience.

Ecological systems can often occur in more than one state that have qualitatively different community structures and rates of ecosystem processes, and which provide dissimilar ecosystem services^1^. Transition to an alternative community state can occur rapidly^2^, with many such abrupt shifts reflecting a non-linear response to a gradual environmental change when a threshold value (‘tipping point’) is exceeded^3^,^4^,^5^. Abrupt shifts are difficult to predict^1^,^6^, and when one occurs, it almost invariably is unexpected^7^ despite the often enormous ecological and social consequences^3^,^8^. There is considerable attention currently focused on means to forecast abrupt state changes, understand how readily they can be reversed, and identify ways to prevent persistent shifts to undesired states. Although some of the drivers and underlying mechanisms of state change in various ecosystems have been identified^1^,^2^,^4^, the processes involved and the potential for different outcomes are still poorly understood. Furthermore, ecologists and resource managers interested in evaluating or managing ecological resilience generally lack empirical tools to assess unambiguously whether a given natural system is likely to show a non-linear response to a small change in an important driver, or if such a threshold exists, to quantify where the system currently lies in relation to that tipping point.

Coral reef ecosystems have been observed to undergo dramatic and sometimes abrupt shifts in community state from one dominated by reef-forming coral to one where other space holders predominate^9^,^10^,^11^,^12^,^13^,^14^,^15^. The alternative space holder often but not always is fleshy macroalgae^11^,^16^, and rapid transitions from coral- to macroalgae-domination have been observed on tropical reefs worldwide, and particularly in the Caribbean. However, coral reefs do not always undergo a persistent shift to macroalgae even following the wholesale loss of live coral from an acute perturbation (e.g., cyclone, bleaching, outbreaks of coral predators). Many reefs, particularly in the Indo-Pacific, regain coral cover on a decadal or less scale after such an episode without transitioning to a macroalgal state^15^,^17^,^18^,^19^,^20^,^21^,^22^,^23^. Low cover of macroalgae appears to be a necessary condition for colonization of coral propagules^12^,^24^, which enables a rapid return of coral following its loss over the landscape. In general, understanding the mechanisms that prevent a shift to an undesirable community state and knowing how close a system is to a tipping point are critical to our ability to manage resilience and sustain ecosystem services.

A key mechanism that has been implicated in coral to macroalgae community state shifts is the loss of top-down control by herbivores^11^,^25^,^26^. Fleshy algae can outcompete corals and inhibit their re-colonization. However, a sufficient level of herbivory can keep standing stocks of these algae below levels at which this occurs^22^,^27^,^28^,^29^. For example, perhaps the best known examples of state shifts have occurred on many Caribbean reefs following decades of intense fishing on herbivorous fishes and the mass die-off of the herbivorous sea urchin *Diadema antillarum*^13^,^30^,^31^. Indeed, overfishing has been argued to be a primary cause of reduced ecosystem function on coral reefs worldwide, with the majority of fished reefs missing more than half of their expected fish biomass^32^,^33^. If herbivore populations are reduced by fishing, then macroalgae may be able to escape top down control on the reef, especially following a disturbance that kills coral on a landscape scale such that herbivory pressure functionally becomes diluted^25^. While numerous small scale experiments where herbivorous fishes were excluded resulted in the proliferation of macroalgae in the absence of herbivory^29^,^34^,^35^,^36^,^37^,^38^,^39^, we generally lack knowledge of the level of herbivory that would be sufficient to control fleshy seaweeds at the landscape scale (but see Graham *et al*.^15^). To date, studies have not explored experimentally patterns of algal development across a gradient of herbivore pressure within a single reef ecosystem. Such an approach could identify whether a non-linear tipping point exists, together with how close the system is to that threshold. The technique could be a potentially powerful tool for assessing key resilience properties within and among coral reef systems, and thus could contribute fundamentally to our understanding of how and why some coral reef systems are more resilient than others.

Reefs on Moorea, French Polynesia have been highly resilient to disturbances in recent decades, with the fore reef returning to coral dominance without undergoing a state shift to macroalgae following perturbations^19^,^35^,^40^,^41^. A recent landscape scale loss of coral^35^,^40^,^42^ afforded an ideal opportunity to assess the level of herbivory needed to maintain control of macroalgae, which potentially could reveal mechanisms underlying the observed resilience of this system. We created an experimental gradient in intensity of herbivory on the fore reef that spanned more than an order of magnitude to quantify the effects of herbivorous fishes on the development of macroalgae and explore whether there was a tipping point. The gradient was established using cages with holes of different sizes to regulate access of herbivorous fishes, resulting in a range in fish herbivory from ambient to none, broadly mimicking effects of fishing. Results from this small-scale experiment were consistent with observed herbivore – macroalgae dynamics at the landscape scale, indicating that our approach can yield powerful insight into key resilience properties of natural systems.

## Results

### Temporal patterns of abundance of herbivorous fishes, live coral and algae on the fore reef

**Figure 1:**
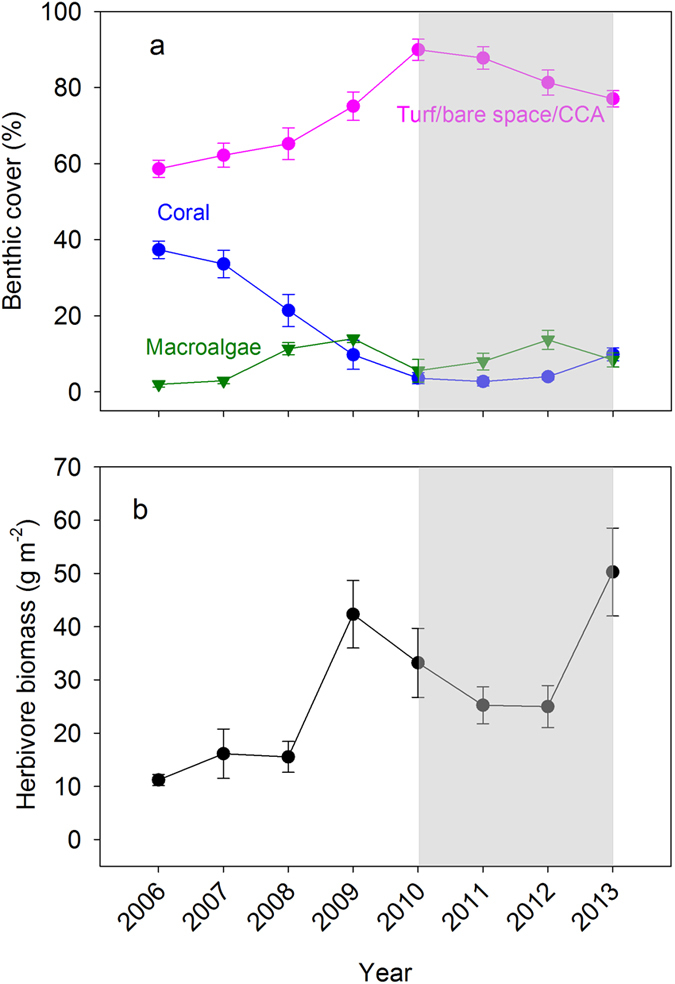
Mean +/− SE (**a**) percent cover of benthic space holders (N = 6 sites) and (**b**) biomass of herbivorous fishes (N = 6 sites) on the fore reef between 2006 and 2013. Shaded area denotes the time period when the experiment was conducted. Benthic cover and herbivore biomass were obtained from MCR LTER core time series data^58^,^59^,^60^. The crown-of-thorns seastar outbreak occurred during 2007–2009, and Cyclone Oli impacted Moorea in early 2010.

Together an outbreak of crown-of-thorns seastar (COTS) and a powerful cyclone (Cyclone Oli) reduced the cover of live coral by >95% on the fore reef of Moorea, from ~35% live cover in 2006 to <5% by 2010 (Fig. 1a)^35^,^40^. The newly-cleared reef provided a substantial amount of substrate for potential algal growth. Despite this, the cover of macroalgae on the fore reef remained low (<15%) and the reef remained dominated by a mixture of crustose coralline algae, bare space and low-growing algal turf during the next several years (2010–2013; Fig. 1a)^35^,^40^. During this time the biomass of herbivorous fishes on the fore reef more than doubled island-wide as their foraging substrate and food supply increased due to loss of coral (mean biomass = 35.2 g in 2009 to 2013 compared to 14.3 g m^−2^ in 2006 to 2008) (Paired t-test, P = 0.0005, t = 8.04, df = 5, N = 6 fore reef sites) (Fig. 1b)^35^,^40^.

**Figure 2:**
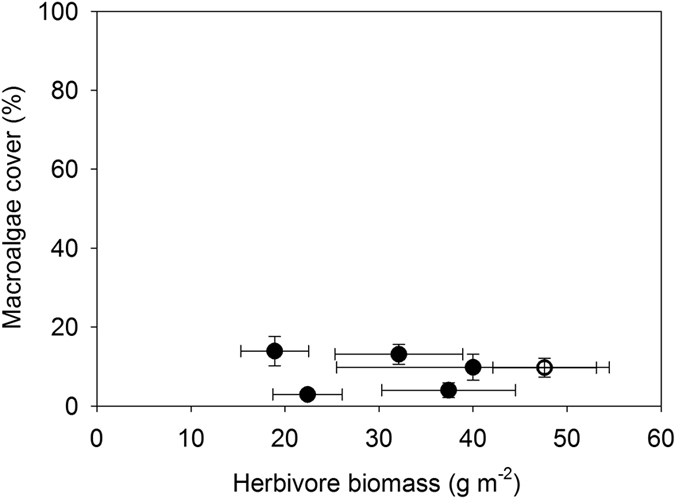
Relationship between biomass of herbivorous fish and percent cover of macroalgae from 2010 to 2013 at each of the six LTER fore reef sites on Moorea. The open circle denotes the reef where the tipping point experiment was conducted. Error bars are +/− 1 SE (N = 4 years).

We explored patterns of herbivorous fish biomass and cover of macroalgae in the period following loss of coral on the fore reef (2010–2013) at six sites around the island. Time-averaged herbivore biomass differed significantly among the sites (ANOVA, F_5,18_ = 3.06, P = 0.036), varying by nearly a factor of three (Fig. 2). However, macroalgae remained low at all sites and was not correlated with herbivore biomass (F_1,4_ = 0.01, P = 0.91, r^2^ = 0.00). These results indicated that on natural substrates at the landscape scale, macroalgae were not able to dominate the substrate over a wide range of herbivore biomass. This finding suggested that any tipping point in the herbivory-macroalgae relationship would fall below the range of ambient herbivory observed among the fore reef sites in the years immediately following the disturbances.

### Estimation of the herbivory-macroalgae relationship

#### Behavioral assessment of the experimental gradient in herbivory

Our multi-year field experiment assessed the development of algae across a gradient of herbivory, and was established along a 225 m stretch of the fore reef at 12 m depth (Supplementary Figure 1). Cages were constructed with holes of different sizes (2.5, 5, 7.5, 10 cm) to regulate access of herbivorous fishes to terra cotta tiles. The assemblage of herbivorous fishes at the experimental site was diverse, consisting of 24 species (Supplementary Table 1) from 3 families observed during the experimental period (Acanthuridae, Scaridae, Pomacanthidae). Six species [*Ctenochaetus striatus, Scarus psittacus, Chlorurus spilurus* (formerly known in French Polynesia as *C. sordidus*), *Acanthurus nigrofuscus, Zebrasoma scopas, Naso lituratus*] collectively accounted for >80% of the total herbivore biomass during the experiment (Table 1). The experimental site had the highest biomass of herbivorous fish we observed during the post-disturbance period (Fig. 2). The goal was to create as wide a gradient as possible, ranging from ambient (uncaged) to no herbivory by fishes (2.5 cm hole size) that would mimic the range in reduction in herbivorous fish biomass that could result, for example, from differing levels of fishing intensity.

**Figure 3:**
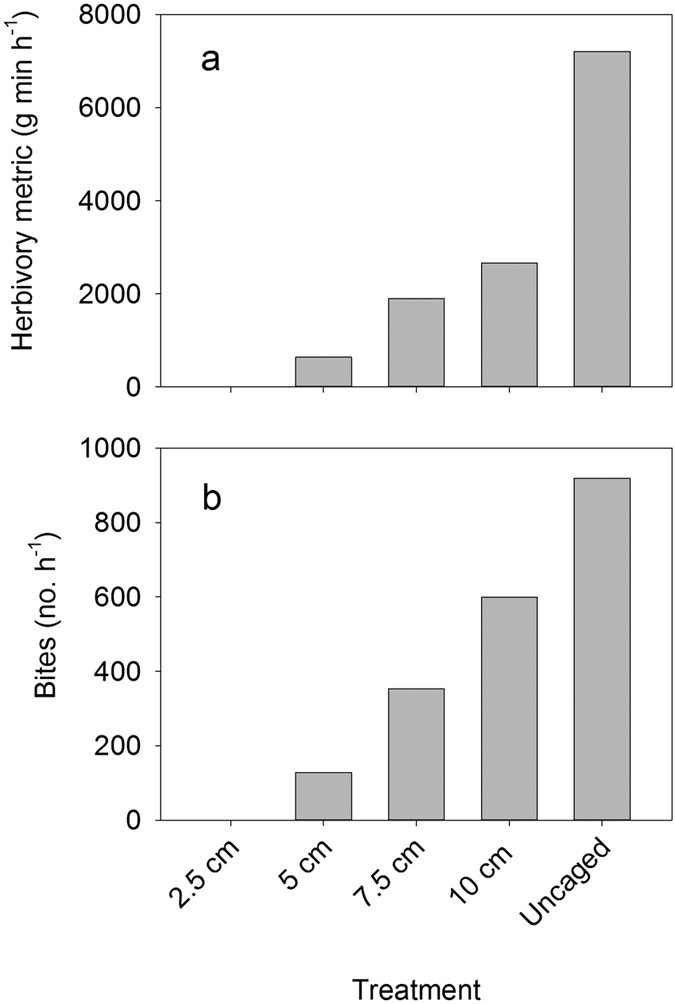
Herbivory metrics for each of five cage treatments based on ~15 hours per treatment of video observations of cages baited with turf algae. Metrics are (**a**) a biomass-based estimate of feeding pressure (g fish min h^−1^) and (**b**) number of bites h^−1^.

Prior to the initiation of the field experiment, we conducted a behavioral assessment to verify that our caging design created a gradient in herbivory. This was done by placing cages of each treatment type in close proximity to each other at the experimental site (12 m depth) and supplying them with fragments of reef substrate covered with algal turf that were collected from the lagoon. Video recordings revealed that herbivorous fishes readily entered and fed from substrates in all but our full cage (2.5 cm mesh) treatments. During the nine-day period covered by the video, herbivorous fishes made a total of 2,139 visits to feed, which resulted in 29,813 bites. We used the aggregated data from the video observations to create a metric of herbivory, and found that the cage design created a very strong gradient in foraging intensity (Fig. 3, Supplementary Figure 2). Although we assessed several metrics of foraging pressure, the patterns were very similar for each. Fish were never observed to enter the full cages with the smallest hole size (2.5 cm). With respect to the remaining treatments, the numbers of visits, bites taken and time (min) spent per hour steadily increased across the gradient in hole sizes and was highest when foraging substrates were fully exposed (uncaged) (Fig. 3, Supplementary Figure 2). Herbivorous fishes of a wide variety of sizes were observed to feed in all the treatments, although the smaller hole sizes precluded entry of the larger individuals (Supplementary Figure S3). To account for the potential effects of variation in size among individuals in our estimate of herbivory intensity, we also calculated a biomass weighted herbivory index (g fish min/hr) (Fig. 3). This metric was similar to the other metrics of herbivory (Fig. 3, Supplementary Figure 2) and was used as our estimate of the intensity of herbivory created by our caging design.

#### Field experiment to estimate the herbivory-macroalgae relationship

**Figure 4:**
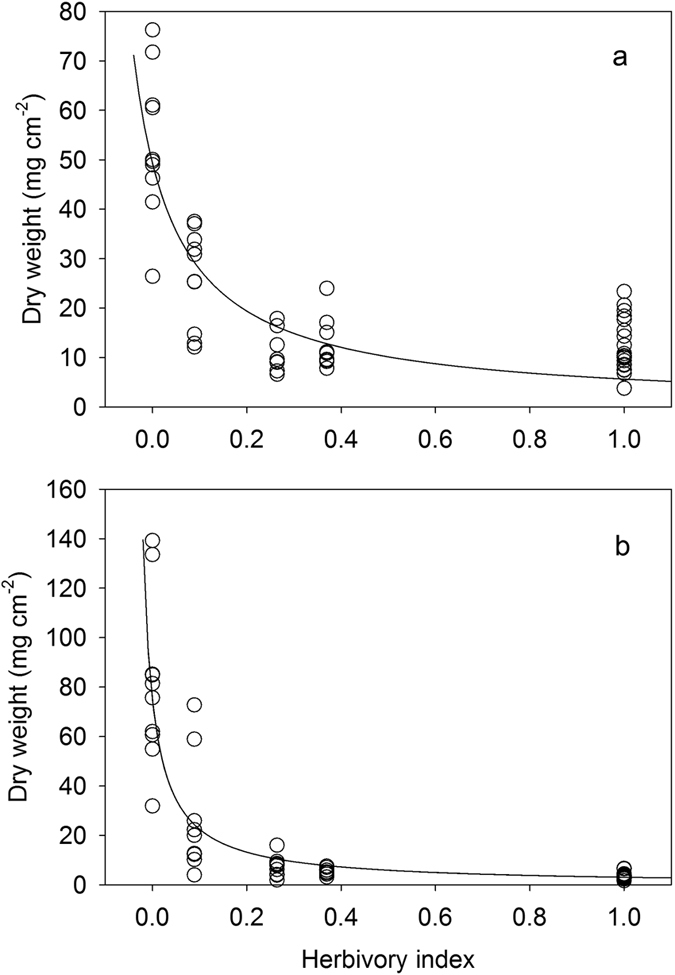
Dry weight of algae that developed on experimental tiles after (**a**) 1 and (**b**) 3 years plotted against relative levels of herbivory. Curves in both plots are the best fit hyperbolic function (for parameter estimates and 95% CIs see Supplementary Table 4). Note that in both years, the biomass of algae increases dramatically once herbivory is reduced to ~10% of ambient. There was a strong effect of experimental treatment on algal biomass after 1 and 3 years (ANOVA, F_5,45_ = 27.58, P < 0.0001 and F_5,45_ = 57.12, P < 0.0001, respectively). N = 10 replicates per level of herbivory.

Our multi-year randomized block experiment (N = 10 replicates) revealed a strong effect of experimental treatment on algal biomass after 1 and 3 years (Fig. 4) (ANOVA, F_5,45_ = 27.58, P < 0.0001 and F_5,45_ = 57.12, P < 0.0001, respectively). The treatment with the smallest openings (full cages, 2.5 cm mesh) had significantly more algal biomass than the treatment with the next smallest openings (Tukey’s test both P < 0.001 after 1 and 3 years). In addition, both of these treatments had significantly more algal biomass than all other treatments (Tukey’s test, both P < 0.001 after 1 and 3 years). The relationship between herbivory and algal biomass that developed after 1 and 3 years was markedly non-linear and was best fit by a hyperbolic function (Supplementary Tables 2 and 3). Total algal biomass remained extremely low and similar on all treatments except for the two lowest levels of herbivory (Fig. 4a,b).

For the two lowest herbivory levels, the full cage treatment that precluded herbivore access had a substantially higher algal biomass (mean  = 53.26 mg/cm^2^, SE = 4.64 mg/cm^2^) than the treatment with second smallest openings (5 cm) after 1 year (mean = 26.16 mg/cm^2^, SE = 3.10), and this differential between the treatments was even more marked after 3 years (Fig. 4; mean = 80.93 mg/cm^2^, SE = 10.59, and mean = 25.16 mg/cm^2^, SE = 7.15 respectively). At the lowest level of herbivory, biomass nearly doubled between the first and third years, whereas it did not change after the first year in the treatment with the second smallest opening (5 cm) that allowed some herbivore access (Fig. 4).

**Figure 5:**
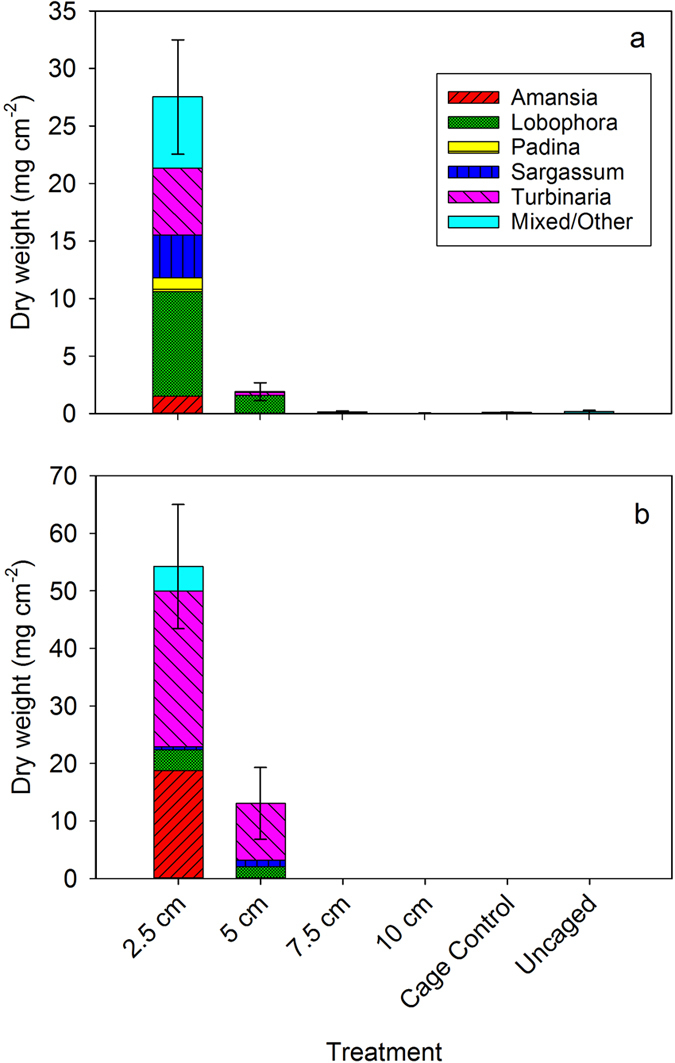
Stacked bar chart showing mean biomass and taxonomic composition of macroalgae present in the different cage treatments after (**a**) 1 and (**b**) 3 years. Error bars represent ±1 SE of the total macroalgae biomass (N = 10 replicates per treatment).

In our experimental treatments, macroalgae did not develop until herbivory was reduced to a comparatively low level. Several taxa of fleshy algae were prominent in the two lowest herbivory treatments, including *Turbinaria ornata, Sargassum pacificum, Padina boryana, Lobophora* sp. and *Amansia rhodantha* (Fig. 5). Indeed, the fully caged treatment was the only one to develop substantial growth of large fleshy algal species such as *T. ornata* and *S. p*a*cificum* at the end of one year (Fig. 5a). These algae did not develop during the first year at the second lowest level of herbivory, and only after 3 years did *T. ornata* occur in any significant amount (Fig. 5b). Together these results indicate that ambient level of herbivory (as experienced by the uncaged and cage control treatments) was more than sufficient to suppress the growth of macroalgae at the study site. The fact that herbivores controlled the development of macroalgae in our experimental cages was mirrored by the rarity of macroalgae on natural reef substrate at both the study site as well as at other sites with significantly lower biomass of herbivorous fishes (Fig. 2). Natural reef substrates and experimental tiles in all but the lowest herbivory treatments were dominated by crustose coralline algae, closely cropped turf, and bare space throughout the experimental period (Fig. 1a).

## Discussion

Herbivory is a key process influencing coral-to-seaweed state transitions on tropical reefs. At the landscape scale, the effects of herbivory have been explored in two ways. The first has been to correlate macroalgal cover with herbivore biomass among reefs that vary in herbivore biomass, either naturally or as a result of differences in fishing pressure, and the second has been to observe responses of reefs following fishing bans^43^. While useful in exposing broad patterns, these approaches can be difficult to interpret due to the many other factors that can affect establishment and growth of algae (e.g., nutrient loading, light, sedimentation, type of herbivores present)^44^. Despite this, non-experimental studies have suggested that the relationship between herbivory and macroalgal cover can be non-linear^15^. At much smaller spatial scales, the effect of herbivory on benthic algae has been examined experimentally numerous times^29^,^36^,^45^,^46^. Experimental explorations generally involve a comparison of ambient herbivory to a treatment where all but the very smallest herbivores (e.g., meso-herbivorous crustaceans) have been excluded; these experiments have, on average, revealed large effect sizes^27^,^28^. In the virtual absence of herbivory, macroalgal cover typically rises rapidly, and after even a few months it can dominate the substrate, particularly on Caribbean reefs^22^. While these studies indicate that fleshy algae often can readily proliferate under conditions of very little to no herbivory, they do not reveal the minimum level of herbivory that would be needed to control fleshy algae. As a consequence, it has not been possible to experimentally evaluate the degree of non-linearity in the herbivory-macroalgal abundance relationship for any given system, or for systems with a marked threshold, to evaluate where ambient levels of herbivory lie in relation to the tipping point.

The experimental design we employed allowed us to estimate a functional shape of the herbivory – macroalgae relationship. The multi-year duration of our experimentally-imposed gradient in herbivory was ample time to capture the response of the macroalgal community^36^. Our experiment revealed a strongly non-linear relationship, and after three years (Fig. 4b) there was a narrow region of herbivory below which macroalgae escaped control. At levels of herbivory greater than this (i.e., around 10–15% of ambient), macroalgae were completely suppressed, overall algal biomass was kept very low, and the community consisted primarily of closely cropped turf and crustose coralline algae. In the treatment below this region (i.e., in the 2.5 cm hole treatment), long-lived fleshy algae such as *Turbinaria ornata* and *Sargassum pacificum* became established by the end of the first year and proliferated further until the experiment was terminated after three years. Importantly, these small-scale experimental results mirrored the qualitative herbivore – macroalgae dynamics on natural substrates we observed at the landscape scale.

In the experiment, dynamics of the algal community at the putative threshold (5 cm treatment) lend further support to the notion that this was the tipping point for control of macroalgae. For this treatment, both turf algae and some foliose algae developed, but the predominant taxon after 1 year was *Lobophora* sp. with flattened thalli, a brown alga that is able to rapidly bloom following loss of coral^20^,^39^. However, after three years, fleshy seaweeds such as *T. ornata* and *S. pacificum* had colonized some but not all of the replicates of this treatment (Fig. 4b), although even when they became established their biomass was kept comparatively low. This is precisely what one might expect to occur in the region of a tipping point where small fluctuations in herbivory can cause a system to ‘flicker’ between alternative community states^6^.

The fore reef of Moorea has historically been highly resilient to periodic disturbances that rapidly kill coral over landscape scales^19^,^35^,^40^,^41^. Over the past four decades, three major perturbation events have occurred on the fore reef of Moorea, each of which reduced the high pre-disturbance cover of live coral by as much as ~95%^19^,^35^,^41^. Despite the sudden, widespread availability of substrata for algae to colonize, the denuded fore reef did not transition to a community where macroalgae were a dominant space holder following any of these perturbations^19^,^40^,^41^. Our experimental and time series data provide insight into a fundamental contributor to this high resilience, namely that the tipping point where herbivores lose control of macroalgae was well below the ambient level of herbivory on the fore reef, at least for the most recent perturbation of 2007–10. Following the widespread death of coral from this event, herbivory pressure was sufficient to maintain the fore reef surface in a ‘non-seaweed’ state suitable for colonization by coral propagules at sites experiencing a wide range of herbivory (Fig. 2). The same may not be true for coral habitats in the lagoons of Moorea where landscape scale death of coral can be followed by a proliferation of macroalgae^47^, or for reefs in other regions where small experimental reductions in herbivory have led to marked increases in fleshy seaweeds^48^.

An ongoing challenge in studies of reef resilience is connecting the findings from experiments to the real world. Our experiment was motivated by several decades of time series data from the fore reef of Moorea that show repeated recovery of coral cover following disturbances so we expected that landscape scale transition to macroalgae would not occur. While these observations characterize well the recent dynamics of the system, they do not reveal the mechanisms preventing a switch to a macroalgae-dominated state. We hypothesized that one important mechanism could be a surplus of herbivory in the system. To test whether there was a surplus of herbivory, we measured algal development on terra cotta tiles across an experimentally imposed gradient in herbivory. By standardizing the substrate available for algal growth and colonization, this approach allowed us to characterize precisely the nature of the non-linear relationship between herbivory pressure and macroalgae, but has the disadvantage that tiles lack the rugosity and structural heterogeneity of natural substrate. One possible explanation for our observation that only a small amount of herbivory was needed to suppress macroalgae, (creating the sharp observed transition, Fig. 4) is the lack of structural complexity of the experimental substrates we utilized (terra cotta tiles), which can make control of algae by herbivory easier. However, the time series data from sites around the island suggest that even on structurally complex reefs, algae were kept under control. In addition, the lack of a relationship at the landscape level between herbivore biomass and cover of macroalgae suggests that ambient levels of herbivory at some sites were likely well above the level needed to control algae, but that a transition to algal dominance is a distinct expectation if herbivore biomass fell to some point below the lowest natural levels we observed (Fig. 2). Of course we do not expect the threshold value of a tipping point to be constant across a heterogeneous landscape, which will be challenging to evaluate from surveys alone but which could be assessed by replicating an experiment such as ours across space.

Dilution of herbivory has been postulated as a potential mechanism underlying transitions to macroalgal dominance on reefs following the sudden, widespread death of coral^25^,^26^. This can occur when a large area of space suitable for macroalgae rapidly becomes available and the capacity of the herbivore assemblage to keep algae cropped over that expanded space is overwhelmed. It is notable that we initiated our ‘tipping point’ experiment immediately following the latest major set of disturbances to Moorea that reduced the cover of live coral on the fore reef from ~35% to ~3%^35^. Thus our experiment was done under precisely the conditions that can lead to loss of control of seaweeds via dilution of herbivory. Despite the fact that our (small-scale) cage treatments were embedded on a vast landscape of space suitable for algal growth, experimentally reduced levels of herbivory were still able to keep macroalgae suppressed until ~10–15% of ambient herbivory was reached. Over the larger, island-wide landscape, macroalgae also were kept under control on the fore reef during the period of our experiment (Fig. 2)^35^,^40^. Since the small bodied sea urchins (median test diameter = 2 cm) on the fore reef of Moorea were not excluded from any of the treatments in the experiment we report here, our results support the conclusion of Adam *et al*.^35^ that herbivorous fishes (primarily parrotfishes) and not sea urchins were responsible for the control of macroalgae on the fore reef of Moorea. One reason why herbivory pressure on the fore reef remained so far above our estimated macroalgae tipping point was a rapid increase in biomass of herbivorous fishes that occurred following the perturbation, likely in response to increased food availability^35^,^40^,^49^,^50^. However, given that we found comparatively little herbivory was required to keep macroalgae suppressed, it could well be the case that even the lower, pre-disturbance herbivory pressure would have been sufficient to keep fleshy macroalgae suppressed. If this was the case, the observed biomass response of functionally important herbivores would strengthen resilience of the coral state without being a necessary condition.

Overfishing of herbivorous fishes has been identified as one major driver of coral-to-seaweed state shifts^11^,^51^. An effect of intense fishing pressure is a reduction in body size of fishes due to size-selective harvesting^52^, and fishers in Moorea target both larger bodied species of herbivores as well as larger individuals within such key groups as parrotfishes^53^. By excluding herbivorous fishes above a sequential series of body sizes in our cage exclusion treatments, our intent was to mimic in a broad sense the effect of variation in fishing intensity on herbivory pressure. Our results show that relatively small herbivores – those with body sizes that can fit through a 7.5 × 7.5 cm opening - completely suppressed macroalgae, whereas even smaller fishes that can fit through a 5 × 5 cm opening were able to greatly suppress seaweed biomass if not entirely prevent their establishment. This contrasts with the notion that large-bodied herbivorous fishes are generally required to control seaweeds^32^,^48^,^54^, but agrees with experimental findings of Cernohorsky *et al*.^29^ for an atoll in the Indian Ocean and Mumby *et al*.^39^ also for Moorea. Of note, the experiment done in Moorea by Mumby *et al*.^39^ that showed no loss of control of seaweeds when large-bodied herbivores (mostly parrotfishes) were removed was a repeat of the same experiment done by this group on reefs in Belize^48^; for the study reefs in the Caribbean, excluding only large-bodied parrotfish led to substantial increases in macroalgae. Thus different sized herbivores can play similar functional roles in different systems, highlighting the need to more fully evaluate the role of variation in functional redundancy in the herbivore assemblage among coral reef systems^16^.

While our study is the first we are aware of that experimentally quantifies the shape of the herbivore – macroalgae functional relationship in a coral reef system, it is likely that such relationships often may be highly non-linear^55^. Differences in the degree to which coral reef systems are close to tipping points will stem from variation in algal colonization and growth rates, the types and biomass of herbivores present and their ability to respond behaviorally and demographically to increased food supply following disturbances^56^. Humans can influence attributes of the herbivore assemblage both directly via fishing^53^,^55^ and indirectly via destruction of nursery habitat for herbivorous fishes^35^. Herbivory alters the death rate of algae, and of course an herbivory threshold should also vary with factors that influence algal birth rates, such as the potential of the reef ecosystem to support rapid colonization and/or subsequent growth of fleshy algae. The latter will depend on the combination of particular algal species present as well as nutrification or other factors that enhance algal growth, and it may vary substantially within and among geographic regions^22^,^38^. For example, Moorea, which is in a very nutrient-poor region of the Indo Pacific, may have a tipping point at a lower intensity of herbivory than reefs located in more nutrient-rich locations. Indeed, repeating this experimental approach across systems can build a more general picture of where such tipping points occur and what may underlie observed heterogeneity in responses.

There is an urgent need to develop more effective strategies for managing resilience in general^30^, and for developing means to detect the approach of a tipping point in particular. With respect to the latter, one method has been to search for metrics of early warning of abrupt transitions in time series data^1^,^4^,^6^. While promising, the utility of this approach will be hampered by the lack of long time series of data for most systems of interest, as well as by ambiguity in interpretation of metrics where appropriate data do exist. Experimental approaches such as ours not only can test predictions from time series analyses, but they also can provide insight where time series data are lacking. For example on coral reefs, widespread use of our experimental design could help distinguish between highly resilient reefs and those where implementation of management actions could stave off an otherwise imminent shift to an undesired community state. Such actions could be triggered in cases where foliose algae develop with only a modest experimental reduction in herbivory^48^, indicating that the ambient level of herbivory is just above the transition threshold. There are, of course, issues related to scaling up the results of experiments to predict dynamics of natural systems, including challenges posed by natural landscape-scale heterogeneity and other factors that could alter the position of tipping points. That notwithstanding, the approach presented here potentially is a powerful means to compare key resilience properties across systems and/or through time. The utility of our approach is three-fold: it provides an estimate of the functional relationship between a driver and response, it can identify a tipping point in that relationship, and it can be used to assess where the ambient system lies in relation to that identified threshold. Knowledge of these aspects for any given coral reef system is fundamental to understanding how the ecological processes that underlie resilience operate, and to developing effective management practices to enhance, maintain or restore resilience^12^,^15^,^16^,^57^.

## Methods

### Study site

Moorea, in the central south Pacific 20 km west of Tahiti, is a triangular volcanic high island with a ~60 km perimeter and an offshore barrier reef that encloses a shallow lagoon. The fore reef of the island recently experienced two major disturbances that dramatically reduced the cover of live coral, including an outbreak of coral-eating crown-of-thorns seastars (COTS) during 2007–2009, and Cyclone Oli, which passed the island in February 2010 and removed most of the dead coral skeletons on the north shore that were left from the COTS outbreak^35^,^40^. The large amount of suitable reef substrate that became available following the loss of coral from the shallow fore reef afforded ample opportunity for the establishment of foliose algae.

### Temporal patterns of abundance of herbivorous fishes, live coral and algae

Temporal patterns of biomass of herbivorous fishes and the cover of live coral and foliose algae on the fore reef of Moorea were assessed before, during and after the disturbances caused by the COTS outbreak and Cyclone Oli. The Moorea Coral Reef Long Term Ecological Research project has collected data on the abundances of fishes annually since 2006 58,59. Annual surveys of all mobile taxa of fishes observed are recorded by SCUBA divers on four replicate 5 × 50 m permanent transects that extend from the surface of the reef to the surface of the water column during the Austral winter at 6 sites on the fore reef (two on each side of the island). The abundances of all non-mobile or semi-cryptic taxa of fishes then are counted along the same transect lines using a transect width of 1 m. The total length (TL) of each fish observed is estimated as precisely as possible; typically to the nearest 0.5 to 1 cm for individuals less than 50 cm in total length. Total lengths are converted to fork lengths (FL) when necessary using the formula FL = aTL + b where a and b represent published species specific scaling parameters. Fish biomass (g) then is calculated using the formula w = aFL^b^, where FL is the fish fork length (FL) in cm and a and b represent published species-specific scaling parameters^58^. Estimates of the percent cover of corals and other major benthic substrata are derived from censuses by scuba divers annually in April along five 10 m transects located at each of the six fore reef sites^60^. Additional details concerning sampling protocols can be viewed at: http://mcr.lternet.edu/data.

### Estimation of the shape of the herbivory-macroalgae relationship

In July 2010, we initiated a field experiment that assessed the development of algae across a gradient of herbivory, which was established using cages with holes of different sizes to regulate access of herbivorous fishes. Sea urchins are small and uncommon on the exposed fore reef of Moorea and they contribute little to herbivory there^35^. The goal was to create a gradient that ranged from ambient to no herbivory by fishes that would mimic the range in reduction in herbivorous fish biomass that could result from differing levels of fishing intensity. The experiment consisted of unglazed terra cotta tiles affixed to the fore reef in one of six treatments. Four treatments had tiles deployed inside plastic-coated wire mesh cages (2.5 cm mesh). One of these was a full cage (2.5 × 2.5 cm mesh) and the other three treatments had increasingly larger holes (5 × 5 cm, 7.5 × 7.5 cm, 10 × 10 cm respectively) cut in the mesh to allow different amounts of visitation by herbivorous fishes. Cages measured 37 × 37 × 12 cm and each contained 4 terra cotta tiles (for harvest at 4, 8, 12 and 36 months). The remaining two treatments also had 4 tiles per replicate and consisted of (1) a cage control (with partial sides but no top) to influence flow but not access by herbivorous fishes and (2) a cage bottom that was fully exposed to herbivores to mimic ambient levels of herbivory. The experiment was a randomized block design (N = 10 replicates of each treatment) with the ten blocks spaced ~20 m apart along a 225 m long stretch of the fore reef 1 km east of Cook’s Bay at a depth of 12 m (Supplementary Figure 1). The terra cotta tiles were seasoned in the ocean for three months, brushed clean prior to deployment, and mounted rough side up. None contained visible algae at the beginning of the experiment.

### Behavioral assessment of the experimental gradient in herbivory

Prior to the initiation of the experiment, we assessed the efficacy of our caging design for creating a gradient in the intensity of herbivory. We placed cages of each treatment type (full cage, and cages with the 3 different sized holes as described above), and cage bottoms that afforded full access to herbivores on the fore reef in close proximity to each other at the experimental site (12 m depth) and supplied them with fragments of reef substrate covered with algal turf that were collected from the lagoon. Turf substrates were replenished at least daily, and underwater video cameras were deployed over a period of 9 days (15 hours per treatment) to record the sizes and species of fishes that visited and their behavior, including entry into the cages and feeding activity on the turf-containing substrates. Videos were subsequently viewed in their entirety and the species, body size (TL), time spent and number of bites taken on the deployed substrate were recorded for each visit. The biomass of each fish visitor was calculated based on published length-weight relationships. Total numbers of visits, bites and time spent in the cages per hour were calculated. In addition, a biomass-based estimate of feeding pressure (g fish min/hr) was obtained by multiplying the biomass of each fish visitor (calculated using the visual estimate of its TL) by the time spent in the cage, then summing these values for all the visits in a treatment and expressing the values per hour of video. We used the biomass-based estimate of feeding pressure to calculate an index of herbivory. This was obtained by expressing the level of herbivory in each treatment as a proportion of the maximum observed (on the control (uncaged) replicates). Because herbivore consumption scales with biomass, our calculated index of herbivory takes into account differences among species and size classes in total algal consumption; we assume that it represented the herbivory during the three year duration of the experiment.

Frequent observations by scuba divers throughout the three-year-long experiment that quantified establishment of fleshy algae along the gradient in herbivory further supported the findings from the video analysis; herbivorous fishes were never seen inside the full cages (2.5 cm hole size), but were observed visiting and feeding inside the cages of all other treatments, the cage controls and on the completely uncaged tiles. In addition, sea urchins and other invertebrate grazers were almost never found in or near any of the treatments.

### Benthic community response to the gradient in herbivory

The biomass of algae that grew on the tiles was quantified after 4, 8, 12 and 36 months (July 2013). We report here results after one and three years of deployment. During each sampling period, one tile from each replicate was transported to the laboratory in seawater and the biomass of algae on each tile was estimated as follows. First, all tiles were rinsed to remove loose sediments. Second, all fleshy algae present were removed, identified, and damp weighed. Subsamples of each algal species were weighed damp and dried in a drying oven until they reached a constant weight so we could obtain relationships between damp mass and dry mass. Next, four 2.5 × 2.5 cm subsamples of non-foliose (turf and encrusting) algal growth were scraped from the surface of each tile, dried and then used to estimate mass of turf and encrusting algae on the entire tile. Total dry algal biomass for each of the 10 replicate tiles within a treatment was summed and expressed as mg/cm^2^. We used ANOVA followed by post hoc Tukey’s tests to first determine whether different amounts of algae accumulated in the different treatments. To account for spatial blocking we used a mixed effects model with plot as a random effect and treatment as a fixed effect. Second, to estimate the functional form of the herbivory-algae relationship we plotted algal biomass for each of the 60 experimental tiles against the herbivory index for their respective treatments. We hypothesized that there would be one of two general relationships between the reduction in herbivory we created in our experimental treatments and the development of algae. First, the development of algae could be linearly related to the total level of herbivory, such that a decrease in herbivory would lead to a proportionate increase in algae. Alternatively, algae may remain relatively rare over a wide range of herbivory, increasing greatly only once herbivory is reduced to sufficiently low levels. To test between these two qualitatively different predictions, we compared the fits of one linear and two non-linear models, using the hyperbolic (y = a/(x + b)) and the negative exponential (y = a*e^(−b*x)^) functions. Generalized linear models were fit via weighted least squares to account for heteroscedasticity using the nlme package in the R programing environment. Separate models were fit for algal biomass after 1 and 3 years and the best model for each year was chosen using AIC. There was no evidence that herbivory levels differed between the uncaged treatments and cage controls, so both were considered to have experienced ambient levels of herbivory.

This study was approved by the University of California Santa Barbara Institutional Animal Care and Use Committee (IACUC, Protocol 639), and all experiments and other methods were performed in accordance with relevant guidelines and regulations. Permits for field work were issued by the Haut-commissariat de la République en Polynésie Française (DRRT) (Protocole d’Accueil 2010–2011, 2011–2012, 2012–2013, 2013–2014 to RJS and SJH).

## Additional Information

**How to cite this article**: Holbrook, S. J. *et al*. Coral Reef Resilience, Tipping Points and the Strength of Herbivory. *Sci. Rep.* **6**, 35817; doi: 10.1038/srep35817 (2016).

**Publisher’s note:** Springer Nature remains neutral with regard to jurisdictional claims in published maps and institutional affiliations.

## Supplementary Material

Supplementary Information

## Figures and Tables

**Table 1:** Herbivores observed at the study site during the experimental period (2010–2013).

Family	Species	Proportional biomass
Acanthuridae	*Acanthurus nigricauda*	0.02
	*Acanthurus nigrofuscus*	0.02
	*Acanthurus olivaceus*	0.02
	*Acanthurus pyroferus*	0.01
	*Ctenochaetus striatus*	0.19
	*Naso lituratus*	0.02
	*Zebrasoma scopas*	0.05
Scaridae	*Chlorurus spilurus*	0.25
	*Scarus forsteni*	0.02
	*Scarus globiceps*	0.01
	*Scarus oviceps*	0.03
	*Scarus psittacus*	0.35
	*Scarus rubroviolaceus*	0.01

Proportional biomass is the mean proportion of the total herbivore biomass contributed by each species over the four-year period. Note that species that contributed <1% of the total herbivore biomass have been excluded from the table.

A complete list of proportional biomass of all herbivorous fishes at the study site is in Supplementary Table 1.
