# Supplementary material for: Coral Reef Resilience, Tipping Points and the Strength of Herbivory
Source: Sci Rep. 2016 Nov 2;6:35817. doi: 10.1038/srep35817 (PMC5090207; doi:10.1038/srep35817)
Supplement: Supplementary Information [file srep35817-s1.pdf]

Supplementary Material

Coral Reef Resilience, Tipping Points and the Strength of Herbivory

Sally J. Holbrook[1,2*], Russell J. Schmitt[1,2], Thomas C. Adam[2] and Andrew J. Brooks[2]

[1]Department of Ecology, Evolution and Marine Biology, University of California Santa Barbara, Santa Barbara CA 93106 USA

[2]Coastal Research Center, Marine Science Institute, University of California Santa Barbara, Santa Barbara CA 93106 USA

Table 1. Herbivores observed at the study site during the experimental period (2010-2013). Proportional biomass is the mean proportion of the total herbivore biomass contributed by each species over the four-year period.

Family	Species	Proportional biomass
Acanthuridae	*Acanthurus blochii*	0.001
	Acanthurus guttatus	0.003
	Acanthurus nigricans	0.000
	Acanthurus nigricauda	0.017
	Acanthurus nigrofuscus	0.022
	Acanthurus nigroris	0.000
	Acanthurus olivaceus	0.019
	Acanthurus pyroferus	0.007
	Ctenochaetus binotatus	0.002
	Ctenochaetus flavicauda	0.000
	Ctenochaetus striatus	0.186
	Naso lituratus	0.018
	Zebrasoma scopas	0.050
Pomacanthidae	*Centropyge bispinosa*	0.002
	Centropyge flavissima	0.002
	Centropyge heraldi	0.000
	Centropyge loricula	0.000
Scaridae	*Chlorurus spilurus*	0.251
	Scarus forsteni	0.022
	Scarus globiceps	0.005
	Scarus oviceps	0.032
	Scarus psittacus	0.347
	Scarus rubroviolaceus	0.012
	Scarus schlegeli	0.002

Table 2. AIC scores of competing models relating the intensity of herbivory to the total biomass of algae present after 1 year. Note that the model with the lowest AIC is the best model.

Model	Number of parameters	Log likelihood	AIC	Delta AIC
Hyperbolic	4	-215.88	439.76	0
Linear	4	-228.9	465.8	26.04
Negative exponential	4	-228.7	465.4	25.64
Intercept only	3	-255.64	517.28	77.52

Table 3. AIC scores of competing models relating the intensity of herbivory to the total biomass of algae present after 3 years. Note that the model with the lowest AIC is the best model.

Model	Number of parameters	Log likelihood	AIC	Delta AIC
Hyperbolic	4	-187.04	382.08	0
Linear	4	-220.5	449	66.92
Negative exponential	4	-221.34	450.69	68.61
Intercept only	3	-293.03	592.06	209.98

Table 4. Parameter estimates and 95% Confidence Intervals (CI) for hyperbolic fit (y = a/(x+b)) shown in Figure 4. Correlation between parameters a and b was 0.93 for year 1 and 0.57 for year 2.

Year	Parameter	Estimate	Lower CI	Upper CI
1 year	a	6.4	4.51	8.29
	b	0.13	0.08	0.17
3 year	a	3.21	2.69	3.72
	b	0.04	0.02	0.06

Figure Legends

Figure S1. Map of the study site along the fore reef of Moorea showing the ten randomized blocks of the experiment, each of which contained 6 treatments (shown in different colors within one of the blocks). Each replicate had 4 terra cotta tiles that were harvested at intervals during the three-year experiment (4, 8, 12 and 36 months). Map of Moorea was based on an original image (ISS006-E-39837) provided courtesy of the Earth Science and Remote Sensing Unit, NASA Johnson Space Center (https://eol.jsc.nasa.gov) and was modified using Adobe Photoshop Elements v14.1 and Microsoft PowerPoint 2016.

Figure S2. Alternative herbivory metrics for each of the five cage treatments based on ~15 hours of video observations of cages baited with turf algae. (a) Total herbivore visits, and (b) total time spent inside cage (or above the mesh bottom for the uncaged treatment).

Figure S3. Box plots showing the median sizes (fork length in cm) of the six most commonly observed species of herbivorous fishes that visited each of the four cage treatments. Boxes represent the first and third quartiles, whiskers are 1.5 * the inter-quartile range, and points are outliers that fall beyond this range. Plots are based on a total of 1991 visits (*Acanthurus nigrofuscus* = 262, *Chlorurus spilurus* = 716, *Ctenochaetus striatus* = 199, *Naso lituratus* = 33, *Scarus psittacus* = 69, *Zebrasoma scopas* = 712). Note that no herbivorous fishes were observed entering the cage treatment with the smallest mesh size (2.5 cm).

Figure S1

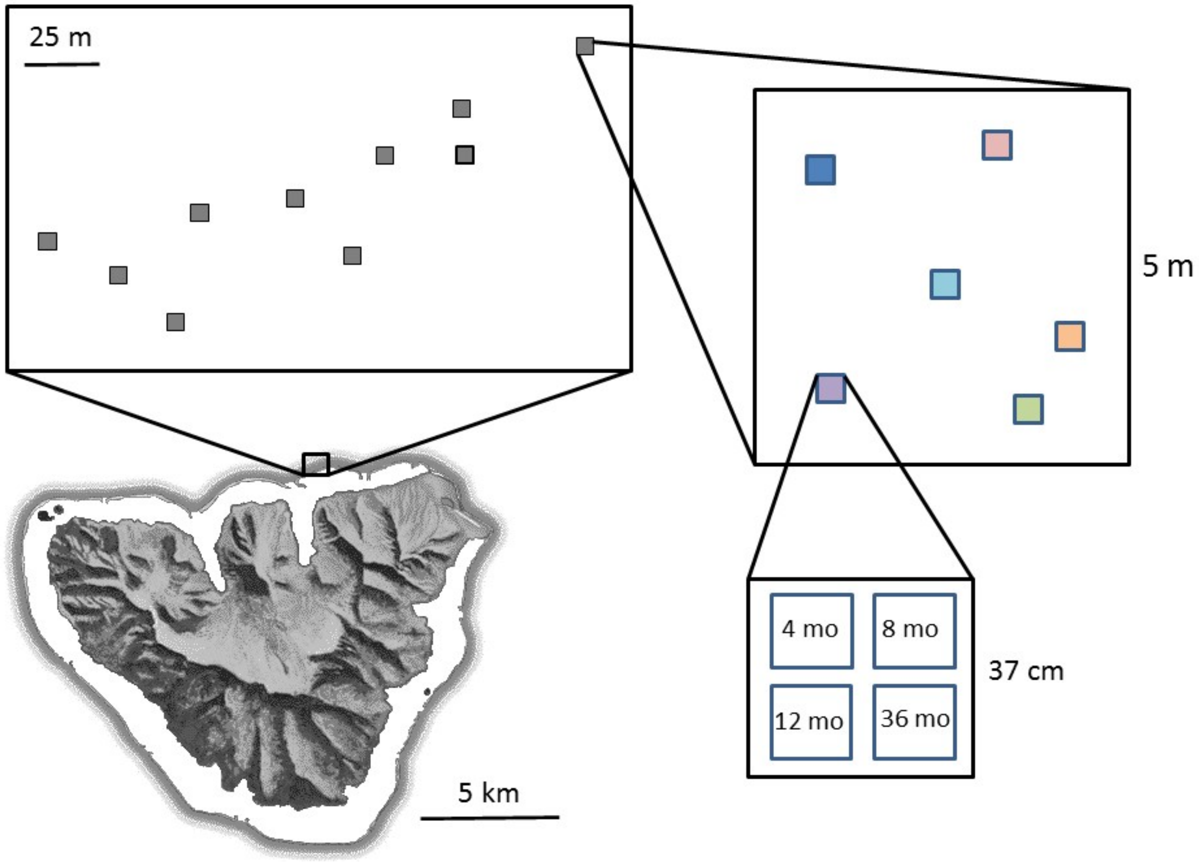

Figure S2

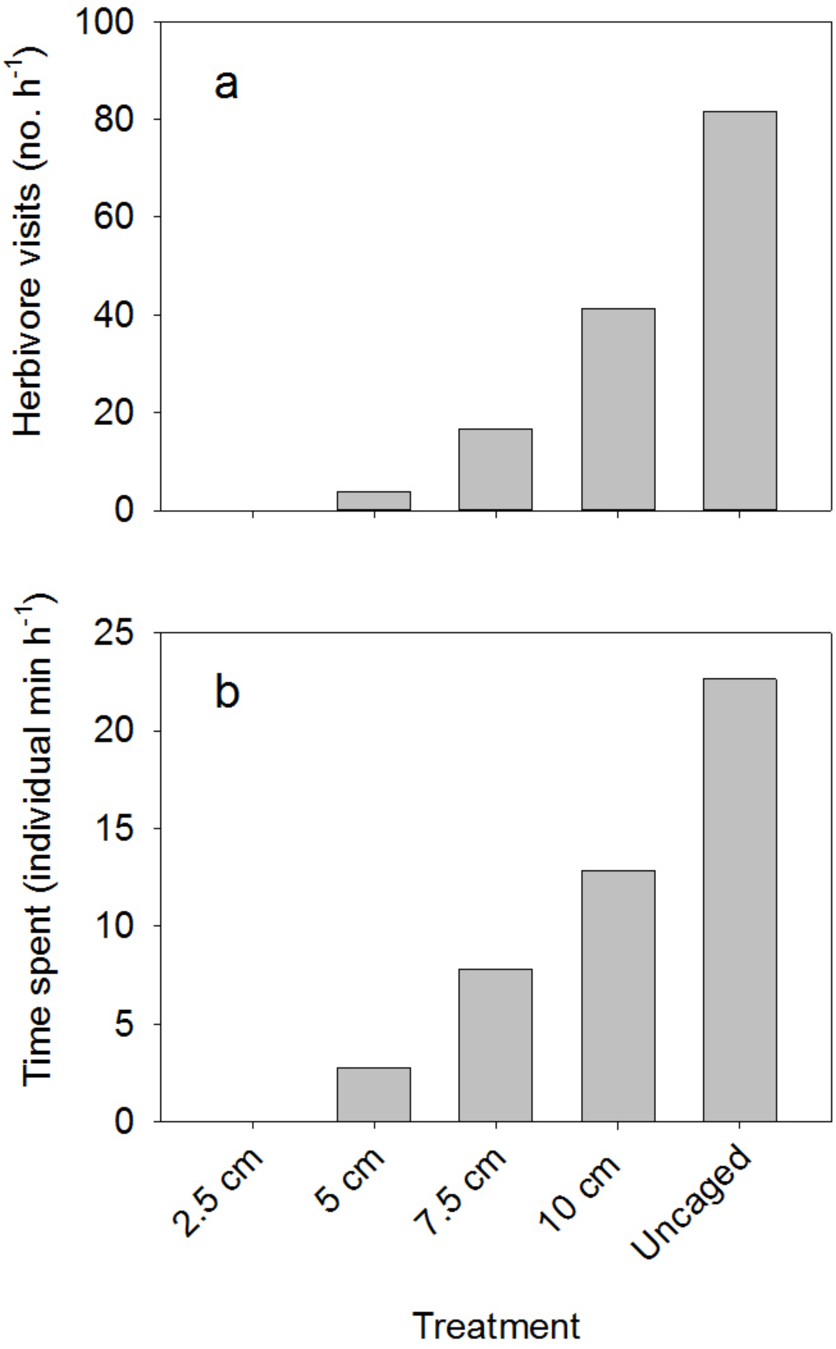
a
b
Herbivore visits (no. h-1)
Time spent (individual min h-1)
100
80
60
40
20
0
25
20
15
10
5
0
2.5 cm
5 cm
7.5 cm
10 cm
Uncaged
Treatment

Figure S3

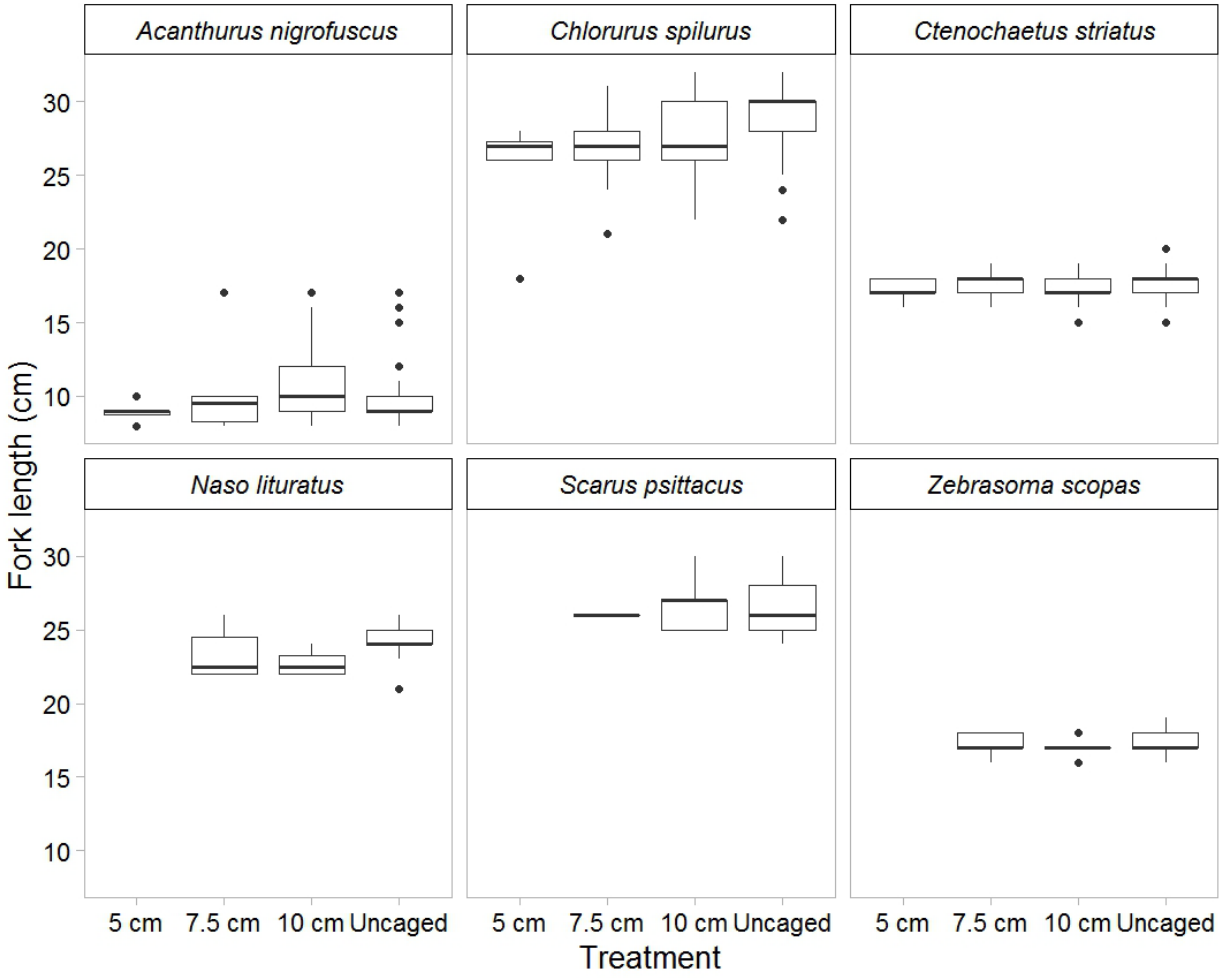

Acanthurus nigrofuscus
Chlorurus spilurus
Ctenochaetus striatus
Naso lituratus
Scarus psittacus
Zebrasoma scopas
Fork length (cm)
30
25
20
15
10
5 cm
7.5 cm
10 cm
Uncaged
Treatment
